# Prevalence and Burden of Refractive Errors at National and Sub-national Levels in Iran

**DOI:** 10.18502/jovr.v17i1.10173

**Published:** 2022-01-21

**Authors:** Seyed Mohammadi, Farshad Farzadfar, Parinaz Mehdi Pour, Elham Ashrafi, Alireza Lashay, Bahram Mohajer, Mohsen Asadi Lari

**Affiliations:** ^1^Translational Ophthalmology Research Center, Farabi Eye Hospital, Tehran University of Medical Sciences, Tehran, Iran; ^2^Non-Communicable Diseases Research Center, Endocrinology and Metabolism Research Institute, Tehran University of Medical Sciences, Tehran, Iran; ^3^Department of Epidemiology, School of Public Health, Iran University of Medical Sciences, Tehran, Iran

**Keywords:** Burden, Disability-adjusted Life Years, Iran, Prevalence, Refractive Errors

## Abstract

**Purpose:**

To estimate the prevalence, burden of refractive errors and their associated trend from 1990 to 2018 and geographic inequalities in Iran.

**Methods:**

Data regarding the epidemiology of refractive errors was extracted from three different sources: systematic review of published literature, data from visual school screening programs, and data from Iran's national health survey (NHS). The pool of all available data on refractive errors as well as demographic, location, and socioeconomic status covariates were fitted in spatio-temporal and Gaussian process regression models to predict the prevalence of refractive errors from the years 1990 to 2018 in 31 provinces grouped by age and sex in order to calculate years lived with disability (YLDs).

**Results:**

In 2018, the age-adjusted prevalence of refractive errors was 16.32% (95% uncertainty interval [UI]: 12.44–21.48%) in both sexes, 17.98% (95% UI: 13.74–23.61%) in women, and 14.66% (95% UI: 11.14–19.36%) in men. The prevalence of refractive errors reveals that it increases with age. Refractive errors contributed to 441.41 and 348.38 YLDs in men and women, respectively. The age-standardized prevalence growth was 31.30% in females and 24.32% in males from the years 1990 to 2018. Significant geographical heterogeneity was observed. The age-standardized YLDs rates of refractive errors represent an increasing trend of 28.9% increase from 1990 to 2018.

**Conclusion:**

Over 28 years, the prevalence of refractive errors increased significantly. Women tend to have higher rates of prevalence. The prevalence increased in older ages. Border provinces had the lowest prevalence. Age-standardized YLDs rates of refractive errors increased by about 30%.

##  INTRODUCTION

Refractive errors are recognized as approximately half of the causes of visual impairment (VI) and the second leading cause of functional blindness.^[1]^ It impinges on patients' quality of life through functional, psychosomatic, and cosmetic issues while also causing economic burden.^[2]^ Refractive errors are listed among one of the four non-fatal disorders classified in the 20 top causes of disability-adjusted life years (DALYs).^[3]^ Since refractive disorders mostly initiate early in life, remarkable morbidity assessed by years of living with disability (YLDs) is associated more with this eye disorder as compared to other ocular diseases.^[3]^ Compared to other causes of VI, refractive errors in most cases is easily treatable by prescribing glasses which is one of the most cost-effective interventions in eye care. If left uncorrected, refractive errors can affect performance, reduce employability, and productivity, and compromise the entire life of patients.^[2,3,4,5]^

Uncorrected refractive disorders relative to the total DALYs increased by 42% globally when compared to 1990,^[6]^ and refractive disorders were responsible for the loss of healthy life of approximately 44.8 years per 100,000 population globally with an increasing trend and with an advance in age from 40 years onward.^[7,8]^ The Eastern Mediterranean region has the second-highest DALY recorded from refractive errors among world regions. This is the consequence of a higher prevalence (188.7; 95% uncertainty interval [UI]: 125.3–276.9%) of refractive error in addition to the suboptimal implementation of prevention/treatment options. Therefore, cost-effective prevention programs are recommended to address this epidemiological priority.^[6]^ Refraction/accommodation (functional) disorders in Iran accounted for 0.42% DALY in 1990 and 0.47% in 2010, where the associated costs and issues were greater than that of other causes of VI, namely cataract, glaucoma, and macular degeneration.^[9]^

Providing reliable evidence and situational analysis is required for promoting good health practices. Global Burden of Disease (GBD) 2017 has presented estimates for the economic and social burden of refractive errors; which, however, lack subnational estimates and comprehensive inclusion of the present national surveys data in Iran.

This study aims to estimate the incidence, prevalence, burden, and trend of refractive errors during the period of 1990 to 2018 at the national and sub-national levels in Iran.

##  METHODS

### Data Sources

**Figure 1:**
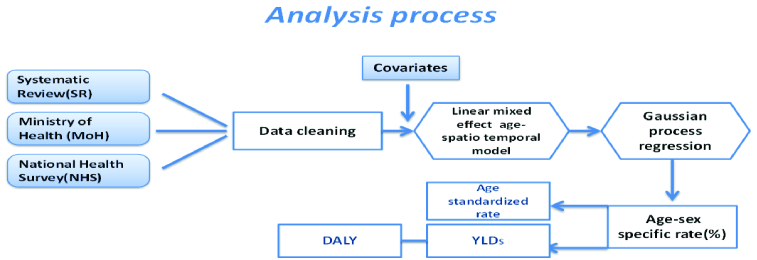
Analysis process for YLDs calculation. DALY, disability-adjusted life years; YLD, years lived with disability.

This research represents secondary data analysis on three data sources; the systematic review of published literature, data from the Ministry of Health and Medical Education (MoHME) screening programs, and Iran's National Health Survey (NHS) data [Figure 1]. A detailed explanation of the search strategy and cleaning process of all data sources is presented in a supplementary document.^[10]^

#### Systematic review

Published literature between January 1980 and December 2018 present in Medline (PubMed), ISI Web of Science, Scopus, Iranian Digital databases of SID (http://www.sid.ir), Barakat knowledge network system (http://health.barakatkns.com), and in the national ophthalmic literature database by Noor Ophthalmology Research Center (http://iraneyedoc.com) were all searched. Additionally, the abstract books of the Iranian ophthalmology annual congresses from the years 2008 through 2010 were screened and included only if the inclusion criteria was satisfied. A detailed explanation of the study selection criteria and critical appraisal is described in the protocol paper.^[10]^ A crosswalk method was applied in extracting the pertinent data where cases related to myopia/hyperopia/astigmatism were combined to those of refractive error and modified the rural/urban scope. A total of 146 data points from 10 provinces were extracted from the systematic review and included in the analysis [Table 1].

**Table 1:** Data source specifications.


**Source**	**Sex**	**Age (yr)**	**Province**	**Year**	**Coverage**	**Number of data point**
**Systematic Review**	Male/female	All ages	10	1999–2014	Sub-national	146 (5.23%)
**Ministry of Health**	Male/female	7–15	31	2007, 2010, 2012	National/sub-national	1156 (37.6%)
**National Health Survey**	Male/female	All ages	31	1990, 2000	National	1049 (41.43%)

**Table 2:** Prevalence of refractive errors by sex and age group in year 2018.


**Age group (yr)**	**Sex**	**Prevalence**	**Lower limit**	**Upper limit**
0–6	Female	1.25	1.1	1.41
0–6	Male	1.01	0.9	1.13
7–11	Female	4.26	3.04	5.97
7–11	Male	3.33	2.34	4.75
12–14	Female	7.6	5.56	10.39
12–14	Male	6.1	4.42	8.42
15–17	Female	7.31	5.22	10.27
15–17	Male	5.58	3.91	7.95
18–24	Female	12.32	9.62	15.75
18–24	Male	9.48	7.17	12.54
25–34	Female	11.42	8.69	14.98
25–34	Male	8.53	6.57	11.05
35–44	Female	16.35	12.96	20.66
35–44	Male	13.1	10.12	17.02
45–54	Female	27.16	21.51	34.23
45–54	Male	25.62	19.56	33.56
55–64	Female	30.71	24.55	38.45
55–64	Male	23.12	18.1	29.6
65–74	Female	32	24.99	40.95
65–74	Male	26.43	21.05	33.17
75–84	Female	36.22	26.91	48.93
75–84	Male	27.94	20.57	38.14
85	Female	29.22	20.72	41.31
85	Male	25.75	19	34.95

**Table 3:** Age-standardized DALYs per 100,000 in different years and sex.


**Year**	**Male**	**Female**
1990	144.95	177.86
1995	172.69	213.64
2000	205.87	257.28
2005	255.14	319.81
2010	302.38	381.64
2018	348.38	441.41

**Figure 2:**
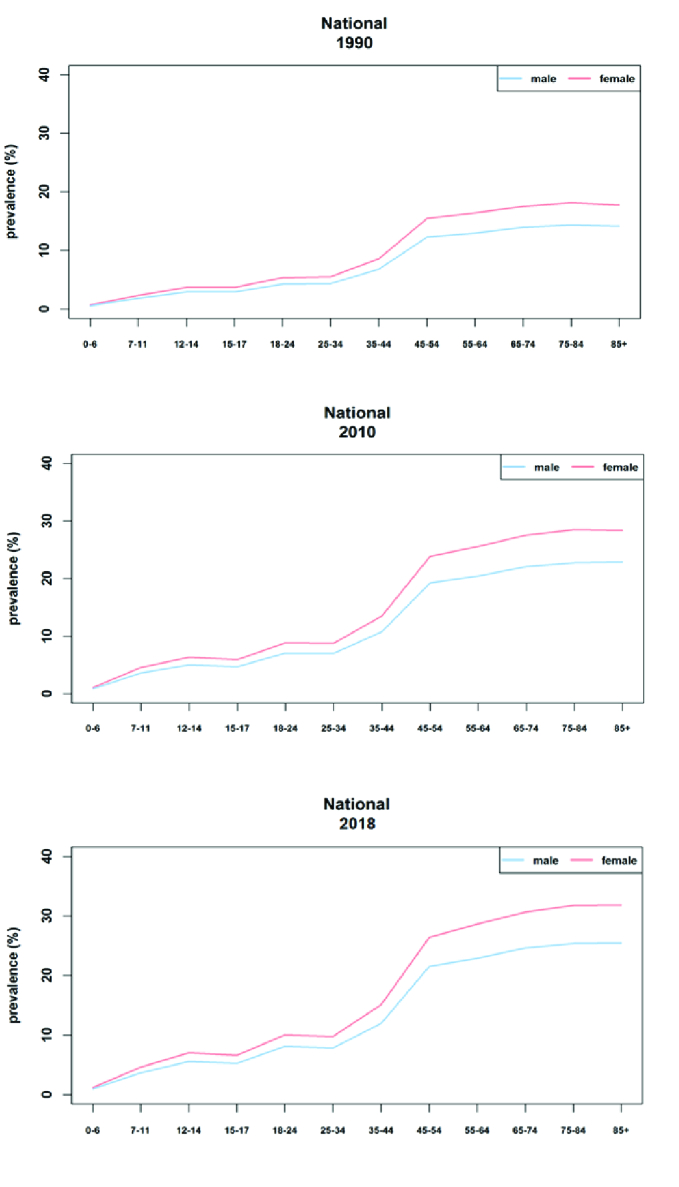
Refractive errors prevalence at national level by sex and age group from 1990 to 2018.

**Figure 3:**
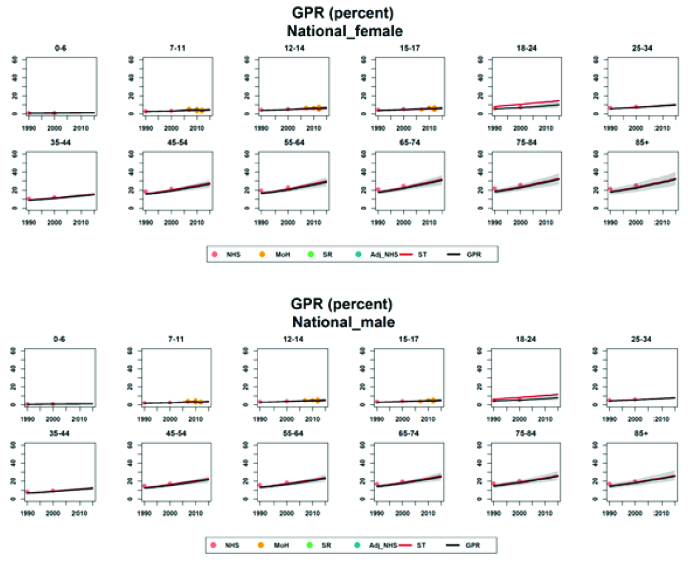
Trend of refractive errors prevalence at national level by sex and age groups from 1990 to 2018.

**Figure 4:**
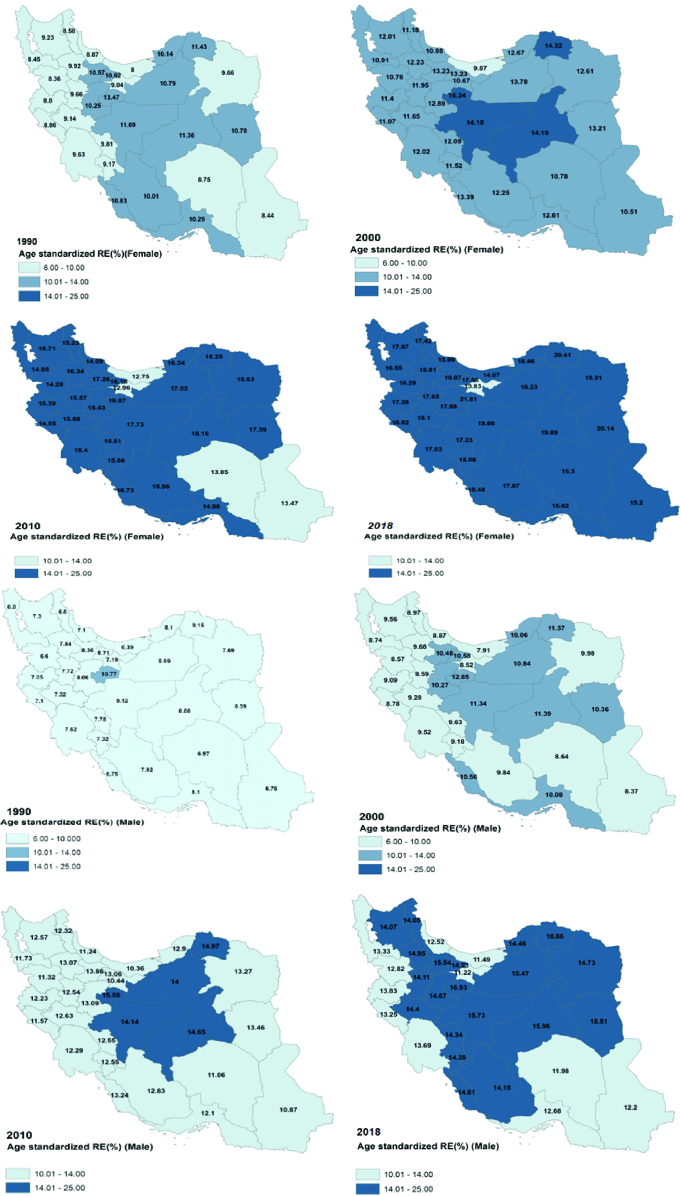
Geographic distribution of age-standardized prevalence in different years (a: Females, b: Males).

**Figure 5:**
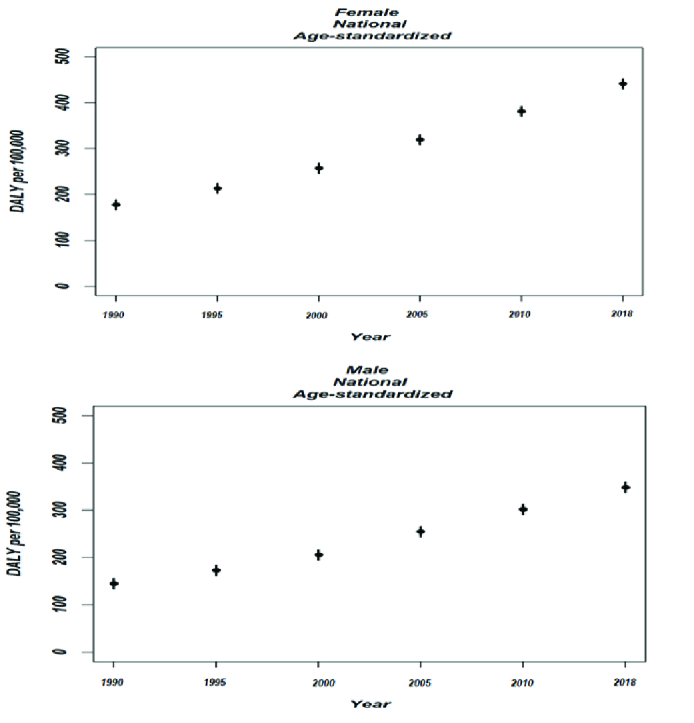
Age-adjusted DALYs (YLDs) per 100,000 at national level by sex from 1990 to 2018.

#### Refractive error screening programs

Unpublished official data for the prevalence of refractive errors occurring in the years 2007, 2010, and 2012 were gathered from screening programs performed in the elementary schools of all the provinces. A total of 1156 data points were extracted [Table 1].

#### National Health Survey

The National Health Survey (NHS) includes two self-reporting questions about spectacles use (“Do you use glasses?”) and spectacles need (“Do you need glasses to see clearly?”) in all age groups and provinces in the years 1990 and 2000. After an adjustment of the NHS data by MoHME/Systematic Review Data while using a crosswalk method and adjusting for missing data, a total of 1488 data points were generated [Table 1].

### Statistical Analysis

A total number of 2790 data points were included from the above data sources. We aimed to estimate the prevalence of refractive errors for different age groups, between the two sexes, at national level, and in 31 provinces, from the years 1990 to 2018. Generalized Linear Mixed Model (GLMM) was applied to impute missing values of these age-sex-location-time combinations. In this model, we predicted the prevalence of refractive errors with fixed covariates, including years of schooling, wealth indices, urbanization ratios, as well as the random effects of the location of the provinces. Urbanization ratios and population data were retrieved from the national censuses, which were conducted by the Statistical Center of Iran (SCI). To calculate other covariates in the statistical models, we used Household Expenditure and Income surveys from the years 1990 to 2018, which were also conducted by the SCI. The second step after applying the GLMM was the application of an age-spatio-temporal (AST) model capturing all variations for time, location (province), and age groups of residuals from the first model. In the AST model, we assumed that there were unmeasured variations in residuals that were derived from GLMM. To estimate these variations, we weighted adjacent years, provinces, and ages by three matrices and used neighboring elements of the matrix that had more correlation with each other where this measure decreased by increasing their distances. The weighted residuals were then added back to GLMM predictions and final estimates of prevalence were produced. In order to have robust estimates with certainty, using estimated rates, we employed Gaussian Process Regression (GPR) which is a Bayesian technique. It defines a flexible model with hierarchical priors for its parameters and it also has a mean and covariance function. In this study, these functions were defined in the AST model and the Matérn function, respectively. Gaussian Process Regression (GPR) samples were drawn from the posterior distribution by using the Markov Chain Monte Carlo method and we calculated the median for the final estimates of prevalence and 2.5 and 97.5 percentiles for its uncertainty.^[10]^

The data collected from the 2018 record of the national population in Iran was used as the standard population in a direct age-standardized analysis to facilitate the statistical comparisons between provinces.

Since refractive errors do not have fatal consequences, DALYs were considered equal to YLDs, which were estimated by multiplying the prevalence by the reported disability weight for refractive errors in the GBD study, and the duration of symptoms.

##  RESULTS

### Prevalence of Refractive Errors

At national level, in the year 2018, the age-adjusted prevalence of refractive errors was estimated at 16.32% (95% UI: 12.44%, 21.48%). It is 17.98% (95% UI: 13.74–23.61%) in women and 14.66% (95% UI: 11.14–19.36%) in men. The age-standardized prevalence increased by 31.30% in females and 24.32% in males during the years 1990 to 2018.

The prevalence of refractive errors showed an increasing trend with age in both sexes, with the highest prevalence among women older than 85 years with a prevalence of 29.22% (95% UI: 20.72–41.31%) in 2018. The highest rise is seen in ages 35 to 44 years [Table 2; Figures 2 & 3].

### Geographic Distribution of Refractive Errors

The age-standardized prevalence of refractive errors in different provinces is reported in Figure 4. Provinces heterogeneity was observed consistently during the study period. The highest age-standardized prevalence recorded in 2018 was 20.4% in North Khorasan while the lowest was discovered in Tehran with a prevalence of 13.8%. Country provinces located at the borders tend to have lower levels of refractive errors while central provinces showed higher levels, particularly in recent years. The data also show a negative association with the wealth index (*r*: 0.6; *P*: 0.032).

### The Burden of Refractive Errors (Per 100,000)

Refractive errors contributed to 441.41 and 348.38 YLDs in men and women, respectively. The YLDs in 2018 in age and gender groups are summarized in Table 3. The highest number of years of life lost was seen in women over 85 years with 499.55 YLDs.

Nationally, the age-standardized YLDs rates of refractive errors reveal an increasing trend of 28.9% during this period [Figure 5].

##  DISCUSSION

This study represented a national first effort to collect data from multiple sources at the individual level to estimate the prevalence of refractive errors over an extended period in Iran. It revealed that a total of 13 million Iranian citizens are affected by refractive errors. Similar to the global patterns, advancing age, female gender, and lower socioeconomic status were identified as risk factors.^[6,11]^

Refractive error prevalence carries an inherent “age-related” feature; where it was determined through the study that the possibility of developing some kind of refractive error increases with age. While in viewing the data of the decade one age category a prevalence of under 10% refractive error was noticed in 2018 throughout the country, it increased to approximately 17% in the ninth decade age category. Several phenomena may explain this, juvenile myopia generally commences before 6 years of age and continues to occur and increase in severity up to 16 years or more. In later years, keratoconus incidence in the second and third decades contributes modestly to increased refractive error. In later years especially where people approach their 40s, presbyopia and latent hyperopia may develop and manifest in patients. The incidence of the presbyopia and hyperopia conditions fluctuates to a higher level during the middle aged category of the population which is clearly reflected in the study diagrams. Decoupling of lenticular astigmatism (from corneal astigmatism) and emergence of nuclear sclerosis may also contribute to the age-related changes. The latter should explain the trend in the > 70 years (index myopia) category. Diabetes mellitus is another source for incidental refractive error in the middle to older ages as it may induce refractive index shifts in the crystalline lens.

The increasing trend in the prevalence of refractive errors is consistent with other reports in the world and region.^[6,11]^ However, our prevalence estimates in Iran are lower as compared with corresponding figures in the region. In addition, relative to global estimates, refractive errors prevalence estimated in Iran reflects a more positive outcome.^[11]^

It is known that the world is experiencing a pandemic of refractive errors.^[12]^ In 2010, about 28% of the world's population was affected by short-sightedness. This is predicted to rise to 34% by 2020 and then to approximately 50% by 2050.^[13]^ The aging population has many more people with refractive errors (demographic transition). Presbyopia and manifest hyperopia are the two major contributors in older ages. The myopia epidemic, on the other hand, may be caused by epidemiologic transition factors; daily habits that encompass the use of digital, sight-intensive, and night and near vision-oriented activities may affect the state of vision in molecularly vulnerable people where they may become more prone to myopia. Many ecological studies suggested a correlation between indoor and nightlife activities with the prevalence of myopia and its related severity.^[14,15,16]^ In addition, lifestyle changes involving the execution of more intense visual tasks such as using a computer or a smartphone led to glasses being prescribed even for patients diagnosed with minimal myopia. An increase in the prevalence of refractive errors in Iran may be attributed to lifestyle changes due to technological advancements and behavioral factors relating to specific age groups. The initial and progressive pace of juvenile myopia in recent years were both attributed to near-work activities and digital life. Cohort studies have proven the axial growth in the eyes in the global population.^[17]^ The COVID-19 pandemic due to the lifestyle restrictions has exacerbated the use of digital apparatus and the need for indoor entertainment where now there are frequent studies revealing more myopia incidence and progression.^[18]^ However, it should also be mentioned that based on particular studies this phenomenon seems to not be consistent among all races, Asians being the most sensitive while Africans seem unsusceptible.^[11]^ The age-standardized prevalence estimates of refractive error in our study from the years 1990 to 2018 translate into > 100% rise which mostly follows the trend of juvenile myopia.^[13,19]^

The authors of the current study would like to indicate the challenge experienced in the conducting the assignment of the disability index for VI to refractive errors. Refractive errors are highly heterogeneous and their severity is divergent. Although some refractive errors are essentially a variation of normality, others are so disabling that without correction they constitute clear handicap and “functional” blindness. As a result of our analysis, we recommend that the approach to addressing the global burden of this disease needs major improvement in this regard including cost estimation and social attribution of the disability.

DALYs attributed to refractive disorders increased 52% worldwide as compared to 1990 and increased by 82% in Iran.^[9]^ Our study showed a higher prevalence and DALY as compared to Institute for Health Metrics and Evaluation (IHME). This might be due to the nature of IHME estimation and imputation of missing data by neighboring data because of security and insufficient evidences from Iran in prior years. Imputation from neighboring countries' evidence may constitute another source of disparity.

In our study, it was revealed that the prevalence of refractive errors in central provinces which generally possess better socioeconomic status (better education and more indoor occupations) is higher than those of the marginal provinces. Prior studies on the prevalence of refractive errors have already confirmed higher myopia prevalence in environments that possess more advanced levels of education, income, and professional occupations.^[20]^ Modern careers tend to be associated with sight-intensive work. In several epidemiological studies^[21,22,23,24,25,26]^ as a consequence of the mentioned trends, increased attention is now directed to addressing unmet refractive error correction and control of myopia conditions.

In summary, the challenging data extraction process in our current study proved useful in determining the prevalence of the refractive errors phenomena in Iran. This study has provided the required evidence in determining the associated burden (economic and social) of these conditions and the basis for the recommendation to the respective health authorities about the necessity to address these issues. Over a span of 28 years, the prevalence of refractive errors has increased significantly in Iran by 16.32% (the age-standardized prevalence growth was 31.30% in females and 24.32% in males from the years 1990 to 2018). Prevalence in females was 3.5% higher than that of males. The prevalence rates increased in older ages. Significant geographical heterogeneity was observed where border provinces possessed a lower prevalence of refractive errors. The age-standardized YLDs rates of refractive errors showed an increasing trend of 28.9% during the period. Lifestyle changes and behavioral adaptions due to technological advancements and other social restrictions continues to perpetuate the onslaught of myopia and other VI issues globally, which further emphasizes the need to manage diagnoses, their prevention and treatment of these related issues.

##  Financial Support and Sponsorship

This work was partially supported by the Tehran University of Medical Sciences under Grant number 24423.

##  Conflicts of Interest

There are no conflicts of interest.
